# Swear off

**Published:** 1897-11

**Authors:** 


### Editorial.

### SWEAR OFF.

An esteemed contemporary says: "When examining wine with the microscope for puss cells, they will be rendered readily visible by adding a little tincture of guiac to the specimen which colors the cells a beautiful blue."

If spirituous liquors have reached the point of adulteration with tabbies in Savannah, it is time for its citizens to take the pledge.

Pussies here rarely require a microscope to render them visible, but as they are nocturnal in their habits, the obscurity is apt to make our aim somewhat uncertain. If tincture of guiac will bring them out any plainer, we will sprinkle it freely in our back yard.

